# Transgender and Gender‐Diverse Individual's Experiences of Openness and Concealment at Work in Sweden

**DOI:** 10.1111/sjop.13103

**Published:** 2025-03-25

**Authors:** Theodor Mejías Nihlén, Tove Lundberg, Matilda Wurm, Anna Malmquist

**Affiliations:** ^1^ Department of Behavioral Sciences and Learning Linköping University Linköping Sweden; ^2^ Department of Psychology Lund University Lund Sweden; ^3^ School of Behavioral Social and Legal Sciences Örebro University Örebro Sweden

**Keywords:** minority stress, non‐affirmation, stigmatization, transgender, work‐related health

## Abstract

The workplace is an important part of many people's lives. Many transgender and gender‐diverse (TGD) individuals have negative experiences of their workplace due to discrimination and cisnormativity. Whether or not to be open about TGD experiences, and the degree of openness, is something many TGD individuals struggle with at work. Openness is related to well‐being and job satisfaction and is therefore important to consider when understanding TGD individuals' work situations. This article examines TGD individuals' experiences of openness and concealment regarding their TGD experience at work. Thirty TGD adults from Sweden participated in online semi‐structured interviews, which were analyzed using thematic analysis. Results show that the organizational climate and physical environment, as well as leadership and human resources, set the stage for an inclusive or excluding workplace for TGD individuals. For the individual, these aspects are taken into consideration when weighing up the risks and advantages of being open about their TGD experience at work. Factors such as work climate, the presence of LGBTQ+ colleagues, and access to safe facilities make a difference in the decision about, and experience of, being open or concealing one's TGD experience at work. Personal values, and a prerequisite to pass or not, affect decisions concerning disclosure and create different challenges in managing working life as a TGD individual. Findings are helpful in better understanding TGD people's situation at work and are of use for work management and policymakers in creating a better work environment for TGD individuals.

**Summary**

- Whether, and to what degree, to be open about transgender and gender‐diverse experiences is something that many transgender and gender‐diverse individuals struggle with in the workplace.
- Leadership, physical environment, and work climate are important aspects that transgender and gender‐diverse individuals often take into consideration when deciding whether to disclose it at work.
- Personal values, and a prerequisite to pass or not, also affect disclosure decisions and create different challenges for transgender and gender‐diverse individuals at work.

## 1 Introduction

Workplaces are central for people to make a living financially and can be an important source of meaning and personal development (Lysova 2019). To achieve such positive effects, workplaces also need to offer an inclusive and supportive environment for all employees (Huffman et al. 2021; Lysova 2019). However, not all employees experience their workplaces as positive. Negative work experiences are particularly common among gender minorities (Cancela, Stutterheim, and Uitdewilligen 2024). In this study, we will use the concept of transgender and gender‐diverse (TGD), which broadly includes gender minorities such as transmen, transwomen, and nonbinary individuals and is a generally accepted concept within this community and researchers in the field (Coleman et al. 2022; Zwickl et al. 2024). TGD is also part of the umbrella term lesbian, gay, bisexual, transgender, and queer + (LGBTQ+) which denotes sexual minorities such as lesbians, gays, and bisexuals, as well as transgender and queer‐identified individuals. The + is added to include other sexual and gender minorities that might have other ways of conceptualizing their identity.

International studies suggest that TGD employees have more negative job experiences than heterosexual cisgender people (Cancela, Stutterheim, and Uitdewilligen 2024). For example, TGD individuals are subjected to discrimination (European Union Agency for Fundamental Rights 2024; Suárez et al. 2022; Swedish Agency for Work Environment Expertise 2022), hostility (Brewster et al. 2014; Rudin et al. 2014), and misgendering (Dietert and Dentice 2009). No studies have been found on TGD individuals' work experiences in a Swedish context similar to our sample. One study did look at hiring discrimination of TGD individuals from the perspective of employers (Granberg et al. 2020) and another on gender expression by cross‐dressers at work (Thanem and Wallenberg 2016). However, public reports from Sweden (Björk and Wahlström 2018; Swedish Agency for Work Environment Expertise 2022) and other Nordic Countries (Young Håkansson 2024) highlight similar negative work experiences for TGD individuals to those documented in international studies.

While the literature on the working life conditions of TGD individuals is growing, a better understanding is needed of what TGD individuals experience as helpful or problematic in the workplace. In particular, more knowledge is needed about the conditions that are truly inclusive and supportive for TGD individuals (Cancela, Stutterheim, and Uitdewilligen 2024), including the ability to feel secure in being open with a TGD experience. Although some international results are partially translatable to a Swedish context, knowledge about local experiences is needed because laws and regulations differ between countries (Swedish Agency for Work Environment Expertise 2022).

Below, research on TGD experiences at work will be presented in relation to the minority stress model. Further, research on TGD individuals' openness and concealment at work will be presented in more detail.

### 1.1 Understandings of Transgender and Gender‐Diverse Experiences in the Workplace

Several theoretical perspectives have been used to explain the unjustified differences in general health outcomes between cisgender and TGD individuals, including the (gender) minority stress model, which has also been used in research specifically focusing on work conditions (Cancela, Stutterheim, and Uitdewilligen 2024; Swedish Agency for Work Environment Expertise 2022).

The concept of minority stress was coined by Brooks (1981) and further developed in the minority stress model (Meyer 1995, 2003). While initially focusing on sexual minorities, the model has been adapted in recent decades to include gender minority individuals (Frost and Meyer 2023; Hendricks and Testa 2012; Testa et al. 2015). The model suggests that minorities are exposed to distal and proximal stressors, which in turn affect health negatively. Distal factors include external events such as violence, victimization, and discrimination, and proximal factors include internalized stigma, concealment, and vigilance due to the expectation of rejection (Frost and Meyer 2023; Meyer 2003). The minority stress model also includes the mediating or moderating role of various coping strategies, social support, and identity characteristics, including identity integration, valence, and prominence (see comment in Frost and Meyer 2023).

Later developments in (gender) minority stress theory have suggested that the distal factors are understood as being caused by cisnormativity, i.e., the social norm assuming that an individual's gender expression is naturally aligned with their assigned gender at birth (Suárez et al. 2022; Yavorsky 2016). Also, the distal stressor of non‐affirmation, that is being invalidated or not being affirmed in one's TGD experience or gender identity, is mentioned as specifically important for TGD individuals (Testa et al. 2015).

While minority stress has been studied in the workplace, most studies explicitly using the minority stress model have focused on sexual minorities (Holman 2018; Velez et al. 2013). However, although to a lesser extent, research has been done using the model in relation to TGD people where specific distal stressors, such as discrimination, have been studied (see, e.g., Cancela, Stutterheim, and Uitdewilligen 2024). Findings from the literature suggest that the workplace is in fact the area where most discrimination tends to occur, compared to other areas in life (Baptista et al. 2023; European Union Agency for Fundamental Rights 2024). A UK study showed that 57.8% of TGD individuals had been exposed to discrimination in the workplace (Rundall and Vecchietti 2010). Another study showed that TGD individuals in Canada were up to 2.5 times more likely to be discriminated against compared to their colleagues (Waite 2021). Studies have also highlighted the possible effects of such stressors, such as the risk of emotional exhaustion (Cancela, Stutterheim, Uitdewilligen, and Hülsheger 2024).

Several studies have also focused on TGD individuals' experiences of proximal stressors, such as increased vigilance and internalized negative stereotypes, at work. Cancela, Stutterheim, and Uitdewilligen (2024) noted in a systematic review that expectations of rejection, concealing TGD experiences to avoid discrimination, making career decisions based on gender setup at the workplace, and internalized transphobia have been reported in previous studies.

While studies building on the minority stress model help to explain differences between cis and TGD individuals in relation to work environment and work outcomes, several commentators have raised concerns about the model (Linander et al. 2024; Tan et al. 2020). One critique is that the emphasis on ‘stress’ in the model reifies an intraindividual understanding of minority stress that centralizes the reactions, coping, and mental health of a specific individual (Tan et al. 2020). This leaves the complex interplay between the individual and the context under‐theorized.

One pertinent example of the complex interplay between an individual and the context is the ability to be open with one's gender identity and/or TGD experience. Drawing on the gender minority stress model (Testa et al. 2015), this is related to the concept of *concealment*, which is considered a proximal stressor, as well as the process of *coming out* or *disclosing* one's TGD experience, which can be important to an individual depending on the prominence, valence, and integration of that person's identity, and not being open can make an individual feel as if they are not valuing their true sense of self (Meyer 2003; Rood et al. 2017). Being open or visible has, however, also been shown to be related to an increased risk for exposure to distal minority stress (Rood et al. 2017; Testa et al. 2015). In fact, many TGD individuals conceal for safety reasons in different social contexts (Rood et al. 2017). While these aspects can be studied separately—each offering valuable insights into the specific stressors and processes involved—it is equally important to examine the complex interplay between them.

### 1.2 Concealment and Openness in the Workplace

Exploring the complex interplay of openness, concealment, and exposure to distal minority stressors is particularly important in work settings. Some TGD individuals, especially those who pass in their gender identity, might not prefer to disclose their TGD experiences and can be content with this decision, as they might experience that not being open about their TGD experience makes them feel more affirmed in their gender identity (Rood et al. 2017; Testa et al. 2015). In these cases, one may question whether this could be understood as concealment, or rather as privacy. Choosing to be open about one's TGD experiences in these cases might then be a matter of developing deeper personal relationships with colleagues at work, by ‘letting’ them ‘in’ to one's life world, rather than ‘coming out’ (Nakhid et al. 2022).

Nevertheless, research shows that TGD individuals often actively conceal their TGD experience in the workplace to avoid transphobia and discrimination (Dietert and Dentice 2009; Mizock and Mueser 2014). One study found that some TGD individuals adapted their gender presentation to conform to a more normative gender expression and did not disclose being TGD; for example, they concealed their transgender history or adjusted clothing and vocal tone (Mizock et al. 2017). In line with the gender minority stress model (Testa et al. 2015), these types of concealment can lead to adverse outcomes for individuals, including heightened feelings of workplace alienation, diminished job satisfaction, and decreased commitment to their roles (Newheiser et al. 2017).

These studies highlight that active concealment is a relational phenomenon. If TGD individuals did not experience or anticipate negative reactions, they would not have to actively conceal their gender identity and/or TGD experience. Similarly, studies addressing the opposite of concealment, i.e., coming out or disclosing one's gender identity and/or TGD experience, show that this also requires active management (Björk and Wahlström 2018). For example, Klein et al. (2015) showed that coming out as TGD is a socially contingent and non‐linear process for many people. Others have demonstrated that disclosing one's TGD experience (or not) is an issue of negotiation in the workplace (Dietert and Dentice 2009), where one strategy is to give hints about one's TGD experience to assess reactions and whether it is safe to come out or not (Yasser et al. 2021). Generally, coming out was experienced as a process involving various steps in disclosing one's identity (Budge et al. 2010). TGD individuals have also been found to be more open in workplaces when the organizational climate is TGD‐positive (Huffman et al. 2021). While coming out is not a straightforward process, being able to be open at work can allow the individual to feel whole as a person in the workplace (Lindholm 2003).

To sum up, drawing on the gender minority stress model and the results from previous studies, one important health‐promoting factor at work is to provide a context where TGD individuals do not have to actively conceal their identity or background but can be open to the extent that they, themselves, find meaningful.

### 1.3 Exploring Openness and Concealment in a Swedish Work Context

Very few studies have been done on TGD individuals' work experiences in a Swedish context, with even fewer studies focusing on openness and concealment. However, in one Swedish report on work experiences among LGBTQ+ people, TGD individuals in the sample reported being the most uncomfortable about being open at work (Björk and Wahlström 2018). In this study, only 31% were open among colleagues, and in contact with third parties, such as clients, customers, pupils, and the like, openness was even lower, with only 18% of TGD individuals being out (Björk and Wahlström 2018). In the total of the European Union 49% of transwomen were open to all or most of their colleagues at work about their TGD identity, compared to 51% being open to only a few or none or all of their colleagues (European Union Agency for Fundamental Rights 2024). Among transmen 40% were open to all or most of their colleagues, and for nonbinary and gender‐diverse individuals the number was 36%. In relation to what is known about openness regarding TGD status in connection to well‐being and job satisfaction, these numbers are a clear indication of an unsatisfying situation for TGD employees in Swedish workplaces. This is particularly concerning in a Swedish context, where legislation requires employers to establish a good, safe, and supportive working environment for all employees where ill‐health is prevented (The Work Environment Act, AFS 2015:4). Guidelines issued by the Work Environment Authority state that developing good interpersonal relationships in workplaces is important to create a good working environment for all (The Work Environment Act, AFS 2015:4). TGD individual's opportunities to be open about themselves, without fear of being negatively treated or victimized, are thus an important work environment issue. In order to establish this, more research is needed on the complex interplay between the individual and the context, regarding the ability to be open with one's TGD experience, including what aspects facilitate or hinder openness.

In the present study, our aim was to explore how TGD individuals in Sweden experience their workplaces in relation to openness and concealment. The specific research question is: What aspects hinder or facilitate TGD individuals' possibilities to be open about their TGD experience at work in Sweden?

## 2 Methods

This paper is based on data collected from two different research projects. The first focused generally on minority stress experiences among LGBTQ+ people in Sweden. This project was approved by the Local Ethical Review Board in Stockholm (2018/330). The second project specifically explored LGBTQ+ people's working life experiences and was approved by the Swedish Ethical Review Authority (2022‐05639‐01).

The data used in this study originate from projects conducted by Queer Psychology in Sweden (QueerPsy), a group of researchers united by the ambition to carry out empowering and progressive research on LGBTQ+ health and well‐being. To achieve this goal, the group maintains ongoing dialogues with organizations representing LGBTQ+ communities.

We have throughout the study engaged in a reflexive approach as recommended by Braun and Clarke (2021). We have engaged in discussions within the research group over how we personally and academically relate to various fields of study within transgender studies. This included reflections on how we bring expertise from various areas of psychology, including clinical, work and organizational, social, and qualitative psychology, as well as sexology. In addition, our positions as researchers are informed by our diverse life experiences, representing a wide range of sexual and gender identities and being part of different communities.

### 2.1 Participants and Interviews

The present study includes interviews with 30 participants. Of these, 20 interviews were conducted in 2018–2020 within a project exploring minority stress experiences in everyday life more generally. We aimed at including a large and diverse LGBTQ sample, relevant for the purpose of the broader study. This sample in total included 29 TGD participants. The 20 participants chosen all reported work life experiences in their interviews. While these data contained important information on working life minority stress, additional data collection was considered necessary for a nuanced in‐depth analysis of work life minority stress. Ten participants were considered a reasonable number. Therefore, ten participants were recruited in 2023 to participate in interviews where the specific focus was on working life minority stress experiences. Participants in both data collections were recruited through ads on social media or contact with NGOs targeting either specifically TGD individuals or LGBTQ+ individuals in general. No remuneration was given to participants.

Among the participants, 12 identified as men—more specifically describing themselves as transman, transguy, man‐not cisman, transmasculine, transsexual‐FTM, and transexual‐transman. Four participants identified as women—more specifically describing themselves as transwoman, woman, transgirl, and female transperson. Also, 17 participants identified within a nonbinary spectrum, more specifically describing themselves as nonbinary, transmasculine nonbinary, nonbinary toward feminine, queer‐trans, non‐gendered, gender‐fluid, and agender. One participant also stated having an intersex variation. Some participants had more than one term to describe their TGD experience. Other minority aspects were not asked for specifically, but some participants mentioned intersecting minority identities or experiences, such as being neurodiverse, having a functional variation, having migration and/or refugee experiences, belonging to an ethnic minority, being polyamorous, being a BDSM practitioner, and also, most participants described belonging to a sexual minority. Fifteen participants had received gender‐affirming care, and four had begun an assessment process for such care. Eight had not sought gender‐affirming care, and in three interviews, no such information was mentioned. Eight participants lived in the countryside or in a small village, six lived in a midsize city, eight lived in a large city, and nine did not mention their place of residence. Participants were aged 17–63 years (mean age = 34 years). They had a large variety of occupations and work experiences, and many described both experiences from their current and from previous positions. The jobs mentioned were: delivery service worker, farmer, healthcare worker, artist, lawyer, phone salesperson, psychologist, librarian, social worker, train attendant, security guard, personal assistant, hairdresser, PhD student, educator, teacher and other childcare positions, elder care worker, manager, counselor, bus driver, software developer, and other IT positions.

All the interviews were semi‐structured. Interviews lasted 42–120 min (mean time = 69 min). All interviews were audio‐recorded, transcribed, and anonymized. All participants have been given pseudonyms in the text. In all interviews, participants were first asked if they were familiar with the concept of minority stress and how they understood it. The participants were thereafter informed of how the researchers define these concepts, to reach a common understanding. In the first project, the interviews were structured around questions of minority stress experiences in general, where the participants were given space to decide for themselves what contexts to talk about, including their workplace. Working life experiences were not specifically addressed by the interviewer but were spontaneously mentioned by 20 participants, who therefore were included in the present study. The interviews in the first project were all conducted by six master's level psychology students, all trained and supervised by the authors of this article who are experts in the field of LGBTQ+ psychology. In the second project, interviews specifically focused on working life and included questions relating to minority stress at work and issues concerning the workplace and colleagues. These participants were also asked about openness concerning their TGD experience at work. All interviews from the second project were conducted by the first author.

### 2.2 Data Analysis

We used a reflexive critical realist thematic analysis, as outlined by Braun and Clarke (2006, 2021, 2023). Critical realism recognizes an external reality but problematizes our ability to access it directly. Rather than assuming that our knowledge is a direct reflection of reality, a critical realist perspective understands all representations of experience as interpretations that are framed within broader social and historical contexts. This perspective was chosen to first focus on each participant's unique interpretation of their experiences and, second, to attempt to interpret thematic similarities across participants' stories.

In line with this perspective, T.M.N. inductively coded all parts of the interviews where any kind of working life experience was mentioned (step 1–2, Braun and Clarke 2006). This was followed by initial thematization, where the minority stress theory, as a theoretical framework, guided a deductive thematization (step 3–4, Braun and Clarke 2006). In this process, T.M.N. and A.M. discussed how our positioning in psychology framed the analysis by using psychological theories and concepts. While using a certain theoretical perspective might limit the analysis (Lundberg et al. 2023), it was clear from the coding that openness and concealment regarding TGD experiences were abundant in the data. As concealment is understood as a proximal minority stressor in the minority stress model, and openness is highly related to proximal as well as distal stressors, this topic was considered important for the analysis. Thus, themes regarding these issues were selected for a more focused deductive thematization. The thematization was also led by the main author, T.M.N. under the supervision of A.M. Preliminary themes were reviewed by M.W. and T.L. and discussed between the authors before final themes were decided (step 5–6, Braun and Clarke 2006), while considering updated recommendations about aspects such as consistency and alignment with research values (Braun and Clarke 2023).

## 3 Results

The analysis resulted in two main themes with three and two sub‐themes, respectively (see Table 1). The first main theme “Setting the stage for (non‐)openness at work” considered the various settings of the organizational climate, the physical environment, and leadership and human resources at work in which openness was experienced as challenging, and which TGD individuals became aware of and took into consideration when making decisions about disclosure. The second main theme, “Navigating (non‐)openness at work”, focused on how openness, or concealment, was managed by participants, and the different dilemmas that emerged, depending on the degree of openness they displayed and the choices about disclosure they made. The quotes used were translated by authors.

**TABLE 1:** Overview of themes and subthemes.

Themes	Subthemes
Setting the stage for (non‐)openness at work	Reading attitudes at the workplace and the Importance of having allies The (non‐)gendering of the materiality of work and the physical work environment The importance of positive leadership and organizational support
Navigating (non‐)openness at work	Navigating forced or chosen visibility at work Strategies in navigating concealment

### 3.1 Setting the Stage for (Non‐)Openness at Work

The experience of the overall work climate, including issues such as the general discourse about politics and minorities, environmental factors, and support from colleagues at an organizational level, differed between the participants. These matters were often important for the experience of whether or not the working environment was conducive to being open with one's TGD experience.

#### 3.1.1 Reading Attitudes at the Workplace and the Importance of Having Allies

Being aware of the general climate at work, in terms of normative attitudes and knowledge about TGD individuals among colleagues, was important for many participants when considering their degree of openness at work or if they should be open at all. One participant considered the personal expression and “style” of their colleagues, such as clothing choices and having, or not having, attributes such as tattoos or piercings, as indicative of their values. This could be understood as reading the degree of cisnormativity in the workplace and potential risk of exposure to distal minority stressors and rejection. Other participants mentioned that they reacted to the presence of racist or sexist terminology at the workplace. Such terminology could be subtle but could still be seen as an indication to the participants of a general lack of openness to non‐normative identities and sometimes even hostility. Some participants described having heard outright trans‐critical comments, and others had met negative attitudes about gay or lesbian people, often from clients, which they interpreted as general LGBTQ+ hostility.

Assumptions about attitudes at the workplace were also based on the wider environment, such as the geographical location. When the risk of being exposed to cisnormativity or trans‐negative attitudes at the workplace was considered high, participants were reluctant to disclose their TGD experience. Some participants working in the countryside assumed that their colleagues and clients might not be knowledgeable about TGD issues or even be hostile towards LGBTQ+ people. Knowledge about popular political parties in the area could also lead participants to make assumptions about the values held among their colleagues. For example, support among colleagues for a nationalist‐conservative party (Sweden Democrats) made one participant feel uncomfortable with being open about his TGD experience because he assumed that these colleagues would hold negative views on LGBTQ+ issues. Another participant, Arien, saw the lack of basic understanding of transgender issues as a reason not to fully disclose their TGD experience at their workplace.

The basic understanding? Among the staff? Mm, yeah it would definitely have been different, then I would have been open for sure, I think. Because then people wouldn't have been afraid of it, like they almost also are now. (Arien, 31, nonbinary, counselor)

Arien feared rejection of their TGD experience due to lack of understanding and possible hostility among the colleagues, and had little hope for support or affirmation. Some participants expressed a desire to work in an environment where colleagues would be more understanding of TGD issues, and thus, where it would feel more natural to be open about one's TGD experience. One participant dreamed of a job in a non‐governmental organization targeting LGBTQ+ issues and assumed that openness would be much easier in such a context. Some thought more education about these issues could be helpful to make their workplaces more TGD friendly, while others thought that changes at a societal level would be needed to make a difference. These participants imagined a less cisnormative space and that it would be more beneficial for them.

Some participants highlighted the presence of other LGBTQ+ employees as essential, and several wished for more queer colleagues. Simon described how a lesbian woman at his workplace had reminded other colleagues about his correct pronouns, if they got it wrong:

And she's helped me quite a lot so that people don't misgender me when I'm not there or when I didn't have the energy, then she was the one who told people “Have you misgendered him again? You shouldn't do that!” Such fun! Yeah, it feels like a very nice confirmation too, it gives you courage too anyway, when you're not fighting alone, but someone thinks it's completely natural to stand up for you. (Simon, 35, transperson, elder care worker)

Simon noted that his colleague's behavior created a positive and more queer‐friendly work climate, and reduced exposure to TGD negative comments. He also found great support in this colleague's presence whenever stressful situations related to LGBTQ+ issues occurred. All in all, being open at work was easier with the support of this colleague, stressing the importance of social support as an important resource against various minority stressors.

#### 3.1.2 The (Non‐)Gendering of the Materiality of Work and the Physical Work Environment

Sometimes material aspects, such as uniforms, facilities, and administrative structures, were features that affected decisions about openness for participants. The physical workplace would also, to some degree, frame opportunities for openness and risks of being outed. Some workplaces did not offer access to non‐gendered spaces for changing clothes or going to the toilet, and thus cisnormativity was expressed through material aspects. Drew, who is nonbinary, described worrying about colleagues' reactions based on which toilet they would use.

They have separate girls'–boys' toilets. And I can find that stressful […] I feel like it's a bit difficult every time I have to go there and make a choice […] what thoughts do they have: “You were in the men's room” or “What did you do in the lady's room?” (Drew, 31, nonbinary, software developer)

For Drew, the gender‐marked toilets at the workplace created a daily distal minority stressor, putting them in a situation where they had to actively manage their way of relating to the binary‐gender norm. Another participant mentioned that there was only one toilet at their workplace for everyone, and this was appreciated by the participant because it meant that the choice of toilet did not become an issue.

Some participants who used locker rooms in the workplace also experienced minority stress and being vigilant related to having to choose either the men's or women's locker room. Others avoided using locker rooms altogether to prevent discomfort or being outed. One participant felt exposed in the locker room because being undressed might reveal their transition process in a way they were not ready for. Another participant, who traveled a lot for work, actively chose openness about their TGD experience, to make sure they would have access to “safe” facilities. This participant expressed a feeling of being forced to be open in order to get their needs met, making the disclosure a reaction to deal with minority stress, rather than a wish in itself to be open with their TGD experience. At one participant's workplace, they had changed from separate to mixed locker rooms, which she felt was much more comfortable because it took away the focus on gender for her.

For some participants, the risk of being outed became a problem when an old name or gender label came up in digital systems. One participant who had asked for their gender label to be changed in the administrative system noted that it had not been permanently changed in all the logs. Thus, their former administrative gender label would pop up and risk outing their TGD background involuntarily. This could be hypothesized to risk causing increased vigilance, a form of proximal minority stress.

For two participants, wearing gender‐neutral uniforms at work influenced their decisions about openness. One person described that he was not able to express his gender identity through his clothes, and thus ran a higher risk of being misgendered, and also had to verbally disclose his TGD experience more often. In contrast, Kalle experienced the usage of the uniform as a reason not to need to come out and still be affirmed in his gender:

There are no women's or men's clothes there, because everyone wears the same uniform. So it hasn't felt necessary to come out at work until now. Because I don't need to… I don't get coded in a specific way. It's a uniform. (Kalle, 41, transsexual FTM, security guard)

Kalle's experience was that, in a uniform, others did not gender‐code him, and, if anything, read him in the way he preferred. He thus felt comfortable and had decided that he did not have to actively disclose his TGD experience. While the risk of being exposed to distal minority stress increased for one person due the uniform, the opposite was true for the other participant.

#### 3.1.3 The Importance of Positive Leadership and Organizational Support

Leadership and other organizational support structures could either facilitate or hinder the opportunity to be open at work. While positive leadership was an important source of social support, a less well‐functioning leadership increased the risk of exposure to distal minority stressors. Some participants expressed comfort knowing that their manager or someone in the HR office would be supportive if they were to be exposed to any form of transphobic behavior. One participant described previous worries about coming out but felt comforted when coming out to their manager had been a positive experience. Another participant, Jonas, said that his manager's support at a small family‐based workplace had directly influenced how his colleagues reacted to him being open.

It wasn't a big thing then, because she's the authority at this workplace, she sets the rules, so if she has your back it's not a thing at all. (Jonas, 33, transman, personal assistant)

Jonas assumed that, thanks to the manager being an authoritative figure in the workplace, his colleagues had followed her lead and accepted his TGD experience. Another participant mentioned that, when she came out, she had experienced a negative reaction from some partners to the company where she was employed. They said that they did not want to work with the company if she was there. While the situation had been stressful for her, her manager had stood up for her by appealing to the discrimination law. The reaction of the boss had been very comforting for her and made the coming out process at work run much more smoothly for her. In this case the social support was very important for the well‐being of her at work as it remedied the effect of the experienced minority stress.

In contrast to the supportive leaders described above, some participants felt insecure about whether management or HR at their workplace would have enough knowledge about TGD issues to understand and support them if needed. For example, one participant did not experience their HR office as being knowledgeable about TGD issues and therefore did not feel comfortable about contacting HR for support if they were to be exposed to negative treatment for being nonbinary. Here, the HR staff was assumed to be cisnormative and incapable of offering social support.

In some cases, participants described situations where managers at the workplace were outright dismissive of TGD individuals and issues related to being TGD. Sometimes, managers themselves exposed the participants to non‐affirmation. One participant had overheard their manager talking critically about TGD people, and Mika was misgendered by the CEO of the company they worked for:

And one of them was our CEO. And he misgendered me twice. […] I didn't dare mention it to him, even though he was quite approachable. I still didn't have the energy. I felt like “Where do I go now? If the CEO doesn't think this is important, why would anyone else care?” (Mika, 32, nonbinary, software developer)

Mika felt that when the CEO was misgendering them it might not be meaningful to try to sustain openness in the workplace. Thus, the exposure to distal minority stressors directly affected Mikas decision about disclosure.

### 3.2 Navigating (Non‐)Openness at Work

Beside the organizational and environmental features of the workplace mentioned above, more intrapersonal factors had an influence. The participants had different prerequisites, some due to physical appearance, and others to internal values about, e.g., professionalism and privacy, affecting how they navigated openness or concealment at the workplace. For example, some had transitioned while remaining employed at their workplace, and physical changes made their TGD experience visible whether openness was desired or not. Others were passing in their identified gender or were read as their gender assigned at birth, giving rise to different types of negotiations about openness. Participants who passed were open with their gender identity, but had to decide whether or not to disclose their TGD experiences. In contrast, those who were read as their assigned gender negotiated openness about their gender identity alongside being TGD.

#### 3.2.1 Navigating Forced or Chosen Visibility at Work

For many participants, experiences of passing or not passing in their gender identity were something that they were constantly aware of and considered in many social situations at work. Nonbinary people reported particular challenges in being accurately recognized and affirmed, due to most people's lack of awareness of the existence of nonbinary genders. This directly relates to cisnormativity and hegemonic binary understandings of gender leading to constant non‐affirmation.

For some participants, the concealment of TGD experiences was not an option because others perceived their non‐normative gender expression, and/or because they could not pass as cisgender even if they wanted to. Some experienced this kind of visibility positively, because it meant not having to actively disclose their TGD experiences. Others felt uncomfortable and uncertain about what people thought of them, and some feared and expected transphobic reactions, which is a proximal stressor.

For some participants, openness was an active choice. Many had chosen to make a clear statement via mailing lists or at a work meeting to disclose their gender identity. This was often well received in the workplace or accepted with few overt reactions. One participant described having been very nervous before coming out and was surprised when their entire work group gave a friendly response. However, openness had, for some, led to negative reactions from colleagues. Another participant said that, when they briefly explained the transition process over e‐mail to their colleagues, they got a non‐affirmative response from a colleague they barely knew who directly questioned the validity of their gender identity.

Sometimes, openness was not so easily achieved, even when efforts were made to explicitly come out to everyone in the workplace. One participant wondered whether their colleagues even remembered that they had come out as TGD, due to repeated misgendering. Another participant tried displaying their nonbinary gender identity with a pronoun tag but did not get an increased affirmation from this. Participants described how being open also involved the process of repeatedly reminding people about one's gender and pronoun. This can be seen as an example of how strongly cisnormativity permeates the social environment, leading to continuous non‐affirmation of TGD experiences, even when TGD individuals are open.

Danny felt disturbed when others seemed uncomfortable with their gender identity, and therefore avoided correcting others when being misgendered:

It becomes, well, this uncomfortable feeling. And that I find it just as uncomfortable as the other person does. So for me it's just, well, like just when the person starts apologizing. That's something you want to avoid. (Danny, 32, nonbinary, train attendant)

Danny had hoped that interactions in which they were being gendered would run smoothly, and that their nonbinary identity would not become an issue, because this made them feel that their gender identity was perceived as a problem or something that had to be dealt with.

For some participants, being open was an important priority. One participant described how they connected openness with “feeling free,” which was an important motivator, and explained that it did not matter too much if some colleagues were not respectful. Here, distal minority stressors were considered less burdensome compared to the potential proximal minority stressors of not feeling free due to concealment.

A participant who worked in healthcare felt that taking on a representative role concerning LGBTQ+ issues was necessary due to the lack of knowledge among her colleagues. She therefore actively chose to take on educational responsibilities. Other participants did not actively educate others on LGBTQ+ issues but thought that being open at work was important for representational reasons and thus had a political motivation for their disclosure. One participant described the importance of others seeing TGD individuals in an everyday context. To him, it was meaningful to show that TGD individuals are around; thus, openness was an important statement in a cisnormative environment. Zackary had also thought about these issues but had chosen to only be partially open at the school where he worked. He did not feel completely comfortable with this decision and felt guilty about not being fully open.

I can feel this obligation that I should be more open to set some kind of good example. At the same time, I personally feel it would be much more uncomfortable. (Zackary, 31, transman, librarian)

Zackary further explained that he had told the school counselor that he could tell TGD pupils at the school where he worked as a librarian that they could come to talk to him if they wanted to, and this had also happened on some occasions. Zackary thus managed his guilt through ensuring partial openness, in a safe and controlled way, with certain pupils.

#### 3.2.2 Strategies in Navigating Concealment

Some participants had chosen not to disclose their TGD experience and were generally being read as their assigned gender at birth. Others who had chosen not to disclose their TGD experience were clearly passing in their binary gender identity. Some participants described, however, how they had to hide parts of their past in conversation, to avoid disclosing their TGD experiences. One participant held back certain information from his colleagues, such as having been pregnant and having played in a girls' sports team. For him, this control of information drained energy, and he felt the need to be constantly aware of what he said about himself in everyday conversations. Thus, this awareness made him more vigilant and led to a heightened proximal minority stress response in everyday life.

Several participants were acutely aware of other people's reactions and insecurities about TGD experiences in various situations. They did not want unnecessary attention, and some chose not to be open because they feared that their TGD experiences would make others feel uncomfortable. At the same time, they often wanted to be affirmed in their identity, to be met with correct pronouns, and respected for who they are, which made this a stressful issue. The concealment led them to experience a lot of minority stressors, such as vigilance and being misgendered. Nevertheless, distal minority stress, such as direct discrimination and harassment, could often be avoided.

Jonas had held the same job twice, once before and once after the transition. When returning to his former workplace after the transition, he encountered several people who did not recognize him and would thus relate to him as if he were a new contact. He described this as being a weird experience and felt that he had almost lost part of his past:

We take a taxi and we've been going with the same taxi guy for many years, like, we know each other. But then I've been gone for a year and came back and he's like “Finally, they've hired a guy at this chicken farm,” or something like that. And I just, it's like I don't exist anymore, I mean all my work experience and all my relations that I built up during those years are just gone for some people because I don't look, or they don't see that it's me. (Jonas, 33, transman, personal assistant)

It was an intense and odd experience for Jonas not to be recognized by people he had worked with before his transition. This also made him uncertain about how to handle the situation and led him to reflect about his degree of openness about his TGD experience.

Many participants found it necessary to hide their TGD experience as a means of ensuring their safety. One participant was worried about coming out when being temporarily employed, feeling that their chances of getting a permanent contract might be lower if they were open as a nonbinary person. Another participant recalled an earlier job where she had been fearful of what might have come from being open as TGD, because a lot of patients at her workplace could be aggressive and transphobic. She took many measures, such as adjusting her voice, clothing, and behaviors, to appear more feminine, to ensure that her TGD experience would remain concealed. The participant reported that these investments took both time and effort, but still felt necessary. Again, some distal minority stressors were avoided, but other proximal stressors, such as vigilance and worry, were commonly experienced due to the concealment. Nevertheless, the avoided distal minority stressors were interpreted as potentially severe and therefore worth the exposure to a higher degree of proximal stressors.

Some participants, such as Arien, described insecurities about their own transition process and/or their gender identity, which made them feel unready to come out.

Because in some way it's like, if I'm gonna be ready for other people's questions, I might want to have answered my own first. Otherwise I just stand there, “how come?”, I have no idea. (Arien, 31, nonbinary, counselor)

Arien felt that they needed time to understand their own process. They felt they needed to have a definite and clear answer about their gender identity before coming out, because being uncertain might not be an acceptable position for others. Here, the integration of the minority identity affects the way stressors are perceived.

Nonbinary participants sometimes concealed their gender identity because they thought their colleagues would have trouble understanding the concept of nonbinary identities. Hence, it would be complicated to disclose their gender. They thought it might cause distress among colleagues and potentially also clients. One participant had decided to officially present themselves as a binary TGD man because this felt easier, even though they identified as nonbinary.

Some participants described feeling generally uncomfortable about discussing personal and private matters and therefore did not want to be open about their TGD experiences in the workplace context. They felt it was a private matter and nothing they were interested in being open about. Still, this sometimes would lead to proximal stressors connected to concealment. Another reason for some participants not to disclose their TGD experience was that they thought it inappropriate based on professional values. Two participants who worked in counseling and healthcare thought that being open about their TGD experience would take the focus away from the client or patient and possibly also be disturbing for the client. One of them thought of it as potentially relationally disruptive if they were to disclose their TGD experience to a client once a counseling relationship had been established. Other reasons participants gave for not disclosing their TGD experience in a setting with clients were fear of transphobic or violent reactions. According to one participant, being openly TGD in their work with young teenage boys would be risky. The fear of the boys being openly transphobic and aggressive had led them to choose concealment in relation to clients.

## 4 Discussion

In this study, we aimed to explore how TGD individuals in Sweden experience their workplaces of what aspects hinder or facilitate their possibilities to be open about their TGD experience at work in Sweden. While being open at work can help the individual to feel whole as a person and be true to oneself (Lindholm 2003; Rood et al. 2017), the results of the present study illustrated how concealment, coming out and being open are complex phenomena, where incentives and motives to be open or conceal one's TGD experience differed depending on contexts and individuals. In line with other research (Björk and Wahlström 2018; Dietert and Dentice 2009; Klein et al. 2015), the results thus highlighted that openness is socially contingent and can vary in degree. For example, many participants were partially open in the workplace depending on context, values, and relationships. The study develops the understanding of the complexity of factors in the minority stress model, e.g., how the proximal stressor of concealment (Meyer 2003) relates to distal stressors (Rood et al. 2017; Testa et al. 2015) as well as identity characteristics and personal values. As such, coming out or being open is not just the opposite of concealment but rather part of a more nuanced and contextual process.

The organizational climate matters for feeling secure to come out (e.g., Huffman et al. 2021). The results showed how TGD individuals used different cues to gain an understanding of the underlying values and opinions that their co‐workers may hold, if this was not explicit. Often, work environments were being read as cisnormative, making disclosure feel like an unsafe option. Sometimes, others than colleagues, such as pupils and customers, would make participants worry about being open as they thought they might have a negative understanding of TGD people. This can be understood as hypervigilance and fear of rejection, described as proximal stressors in the minority stress model—negatively affecting mental health (Frost and Meyer 2023; Meyer 2003). Reading nuances of this climate can also be understood as a form of reflexive work, as shown by Lundberg et al. (2023).

Our results showed it being helpful when facilities at work are not gendered in a binary way, and when administrative structures are adjusted to include nonbinary people and can handle people who transition at their workplace in a smooth way. This would lower exposure to some minority stressors, such as being forcibly outed, and facilitate openness as a free choice. Something like wearing a uniform could also become a way to have focus taken off of or be in line with one's gender expression which could be a positive experience but also negative in the way that it would be harder to express one's gender and thus be affirmed. Many participants also requested better knowledge about TGD individual's lives and various identities at their workplaces, among co‐workers, human resources, and at the leadership level. Such awareness could reduce exposure to distal minority stressors, which is a risk factor for emotional exhaustion (Cancela, Stutterheim, Uitdewilligen, and Hülsheger 2024). In either case, it seems the choice of occupation and workplace does make a lot of difference for the possibilities of visibility management.

According to Young Håkansson (2024), human resources and leadership are important for TGD individuals' abilities to be safe at work. This is also in line with the minority stress model, where social support is presented as a resilience factor (Meyer 2003). Our results provided examples where managers behaved proactively to defend TGD individuals and thus had a positive influence on co‐workers, but also cases where people in leading positions had failed to affirm their TGD employees and thus risked creating an unsafe working environment. In addition, relationships with other co‐workers and clients were also highlighted as important factors to consider when navigating openness, in line with international studies (Cancela, Stutterheim, and Uitdewilligen 2024; Swedish Agency for Work Environment Expertise 2022) and Swedish reports (Björk and Wahlström 2018). Active support from co‐workers made a huge difference for feeling safe and included at work. The quality of a relationship with a specific co‐worker was also mentioned as important in deciding whether to be open with that individual. This is in line with the idea of ‘letting in’ rather than coming out (Nakhid et al. 2022), as being mindful about context and relationship to whom you share your inner world rather than making a general statement of coming out. However, some participants described how their attempts to come out received negative or no attention from their colleagues, showing how invalidation and non‐affirmation added another dimension to the complexity of openness. Our results also indicated that many TGD individuals would be likely to benefit if they had community resources available, such as counseling or peer networks, to support them in their situation at work, as participants wished to have more LGBTQ+ colleagues and access to LGBTQ+‐informed support. This is in line with community connectedness having been shown to be an important resilience factor (Testa et al. 2015). Both formal help in dealing with problems and difficult situations at work, and more informal emotional support could be helpful in strengthening social support.

The fact that one participant chose to be out as a binary transgender person, but not as nonbinary even though this was their gender identity, exemplifies that openness can take many routes and be partial. This was a partial solution to the dilemma mentioned by, e.g., Testa et al. (2015) and Rood et al. (2017) of wanting to be open but also avoid the heightened risk of distal minority stressors that come with openness. In this case, the person clearly wanted their TGD experience to be recognized but chose to self‐present with a binary identity within that spectrum because they thought a transgender male identity would make more sense to their co‐workers. In this way, they avoided some of the distal stressors that they feared being exposed to as nonbinary. This could also be understood as a kind of countering of cisnormativity. However, this strategy failed to challenge the binary norm (Aboim 2020), possibly adding more proximal minority stress on a nonbinary individual. Binary norms make certain types of identities difficult to grasp for many people, leaving nonbinary people with an extensive work even to have their bare existence validated (Lundberg et al. 2023).

The participants' personal prerequisites, preferences, and values also mattered in relation to openness. Wanting, or feeling obliged, to be a representative of TGD communities, and/or broader LGBTQ+ issues, led some participants to consider their degree of openness. The results illustrate that a desire to help the community could sometimes be prioritized over one's own comfort. Community is thus seen as a prioritized and valued factor which is in line with the importance of community connectedness as a resilience factor (Testa et al. 2015). For one participant, potential exposure to distal stressors when being open about their TGD experience was completely overruled by their personal values of feeling free and open—a case where the TGD experience can be understood to be highly prominent and integrated for the participant. This is also in line with openness being an important factor for feeling a true sense of self (Rood et al. 2017). In contrast, others found it unprofessional to be open about their TGD experience. Indeed, the sharing of personal life at work in relation to identity can become quite a complex issue to deal with in relation to openness (Di Marco et al. 2024). This also causes a problematic asymmetry because heterosexual cisgender people's gender and sexual identities are usually correctly assumed even when not mentioned, and their “openness” is not considered as “personal” or “intimate”. In contrast, openness as a TGD person becomes an issue that is thought to affect relationships, working alliances, and so on. This is in line with the complexity of the issue of choosing concealment or openness described by e.g. Nakhid et al. (2022). In some cases, however, a non‐normative gender identity would be assumed by others due to the person's physical appearance and expression. According to some participants, such prerequisites were positive, as they relieved the need for “coming out”, but, of course, this also made it harder or impossible to choose concealment of one's TGD experience if this is what one would have preferred.

### 4.1 Conclusions and Implications for Practitioners and Future Research

Many improvements could be made to enhance TGD individuals' situation in their workplaces, to enable individuals to choose how and if to disclose their TGD experiences with no fear of being exposed to minority stress. In their review, the Swedish Agency for Work Environment Expertise (2022) concluded that a supportive work climate and active organizational work with inclusion policies is important for TGD people's well‐being in the workplace. In Sweden, the employer is also required to take active measures against discrimination in the workplace according to Swedish law (The Discrimination Act, 2008:567).

This study provides some insights important for the understanding of TGD individuals' work lives and challenges in managing openness and concealment. The findings can inform organizations, employers, and policymakers on important considerations, such as the need for TGD‐competent support and flexible administrative structures, as being relevant measures when working to improve the situation for TGD individuals in their work environment. Future studies could focus further on the specifics of what and how organizations could improve in order to facilitate the needs of TGD employees. There is also a need for more research to highlight the complexity of concealment and openness for TGD individuals in different areas of life due to the context dependency of this complexity.

### 4.2 Strengths and Limitations

Some limitations of this study should be mentioned. The recruitment was conducted through posts on social media, which may have led to an overrepresentation of people who have a higher than average degree of community connection and online presence. Also, there is an underrepresentation of transwomen in the sample. Furthermore, the inclusion of participants did not take into account which professions they had. Nevertheless, the representation covers a variety of sectors, which probably means that the results are transferable for a large range of working life experiences among TGD individuals in Sweden.

The fairly large number of participants with varied workplace experiences is one of the study's strengths. Even though the initial interviews did not focus solely on working life, they give a broad picture of a larger number of participants' experiences at work. The fact that working life experiences were brought up to a high degree informed the researcher of the need to explore this area in focused interviews, which in turn gave further depth and richness to the study. The thorough work that the research group members have done in supervising and jointly working on the analysis has made continuous credibility checks of the analysis possible (Elliott et al. 1999).

## Author Contributions

**Theodor Mejías Nihlén:** conceptualization (lead); writing – original draft (lead); writing – review and editing (lead); investigation (lead); methodology (equal); formal analysis (lead). **Tove Lundberg:** funding aquisition (lead); conceptualization (supporting); writing – original draft (supporting); writing – review and editing (equal); project administration (lead); formal analysis (supporting). **Matilda Wurm:** conceptualization (supporting); writing – original draft (supporting); writing – review and editing (equal); funding aquisition (equal); formal analysis (supporting). **Anna Malmquist:** funding aquisition (equal); methodology (lead); writing – review and editing (equal); writing – original draft (supporting); conceptualization (supporting); supervision (lead); formal analysis (supporting).

## Ethics Statement

This study has used data from two projects, both of which have been ethically approved; the first was approved by the Local Ethical Review Board in Stockholm (2018/330) and the second was approved by the Swedish Ethical Review Authority (2022‐05639‐01).

## Consent

All participants have given informed consent.

## Conflicts of Interest

The authors declare no conflicts of interest.

## Data Availability

The data that support the findings of this study are available on request from the corresponding author. The data are not publicly available due to privacy or ethical restrictions.
